# Human embryonic stem cell research, justice, and the problem of unequal biological access

**DOI:** 10.1186/1747-5341-3-22

**Published:** 2008-09-29

**Authors:** Mark S Moller

**Affiliations:** 1 Philosophy Department, Denison University, Granville, OH, USA

## Abstract

In 2003, Ruth Faden and eighteen other colleagues argued that a "problem of unequal biological access" is likely to arise in access to therapies resulting from human embryonic stem cell research. They showed that unless deliberate steps are taken in the United States to ensure that the human embryonic stem cell lines available to researchers mirrors the genetic diversity of the general population, white Americans will likely receive the benefits of these therapies to the relative exclusion of minority ethnic groups.

Over the past five years the problem of unequal biological access has not received much attention from politicians, bioethicists and even many researchers in the United States, in spite of the widely held belief in the country that there is an obligation to prevent and correct ethnic disparities in access to medical care. The purpose of this paper is to increase awareness of the problem of unequal biological access and of the need to do more than is currently being done to ensure that ethnic disparities in access to human embryonic stem cell-based therapies do not arise.

Specifically, this paper explains why the problem of unequal biological access will likely arise in the United States in such a way that white Americans will disproportionately receive most of the benefits of the therapies resulting from human embryonic stem cell research. It also argues for why there is an obligation to prevent these ethnic disparities in access from happening and outlines four steps that need to be taken towards meeting this obligation.

## Introduction

In an article published in late 2003, Ruth Faden and eighteen other prominent scientists and bioethicists drew attention to "the problem of unequal biological access" in human embryonic stem cell research [1]. Faden and her colleagues showed that unless deliberate steps are taken in the United States to ensure that the set of human embryonic stem (hES) cell lines available to researchers mirrors the genetic diversity of the general population, there is a good chance that white Americans will receive the therapeutic benefits of this research to the relative exclusion of minority ethnic groups. These authors recognized the injustice of this outcome and rightly stressed our obligation to avoid it.

In spite of the efforts of Faden and her colleagues (and others [2]) to draw attention to the problem of unequal biological access, the events of the last five years have shown that many politicians, bioethicists, and even many researchers in the United States continue to be unaware of it. During this time, public policies related to hES cell research have been debated and implemented and new discoveries in this area of research have been heralded and dismissed without much attention being paid to how they might exacerbate or mitigate this problem. The following examples illustrate this.

The first one concerns the debate over the Stem Cell Research Enhancement Act (H.R. 810), passed by the United States Congress in 2005 and 2007, and vetoed by President Bush both times. The purpose of this bill was to loosen the restrictions imposed by the U. S. federal policy that limits federal funding to just those hES cell lines in existence prior to August 9, 2001. The bill would have allowed funding for research on hES cell lines created after this date as long as they were derived from "discarded embryos," that is, from donated embryos left over from fertility treatments [3]. What is salient about this debate is that discussions of this act in Congress, in academic journals, and in the media centered almost exclusively on whether someone who opposes hES cell research because embryos are destroyed could consistently agree that it is morally preferable to use discarded embryos for research rather than waste them. What was not discussed much, if at all, is the impact that loosening the restrictions of the federal policy would have had on the problem of unequal biological access. Some of those engaged in this debate did claim at the time that allowing additional hES cell lines to be created using federal funding would extend the benefits of hES cell research to more people, but there was little discussion of whether these benefits would be more equally distributed across the general population.

A second example is the National Stem Cell Bank that the National Institute of Health (NIH) authorized to be established in 2005 at the WiCell Research Institute, a "supporting organization" of the University of Wisconsin – Madison [4]. Faden and her colleagues had argued roughly two years earlier that a just, public hES cell bank was the way to address the problem of unequal biological access. Indeed, their paper was really a call to recognize an obligation to ensure that any banks created address this problem by mirroring the genetic diversity of the American population. And yet, the National Stem Cell Bank was created without any apparent recognition of this obligation. Press releases by the NIH announcing the creation of the National Stem Cell Bank emphasize how it will facilitate research by making the hES cell lines approved for federal funding available to more researchers at significantly less cost [5]. However, these releases make no mention of the problem of unequal biological access even though Faden and her colleagues argued that ameliorating this problem is a strong reason to create such a bank. As of this writing, there is also no recognition of the problem anywhere on the website for the National Stem Cell Bank. There are no links to any discussion of the problem and how the bank may or may not address it. For that matter, there are no links to any discussion of the genetic diversity, or lack thereof, of the twenty-one hES cell lines available to researchers through the site. From the information given on this website one could only have the mistaken impression that the problem of unequal biological access is not a problem at all.

The third example is the initiatives by various states to publicly fund hES cell research that does not qualify for federal funding. California, for instance, has allocated three billion dollars over ten years to support the efforts of in-state universities and institutions to pursue such research. Public funds can be used in California for hES research done with hES cell lines derived from discarded embryos after August 9, 2001, as well as for research on perfecting the use of somatic cell nuclear transfer (SCNT) to create embryos from which hES cells could then be obtained [6]. Six other states have similar initiatives, although none of these have allocated the same level of funding to support hES cell research in their states, and some limit funding to just hES cell lines derived from discarded embryos [7]. What is significant about these initiatives given the present discussion is how state officials have justified the need for them to their constituents. While these officials have certainly emphasized that these initiatives are necessary for ensuring that the full therapeutic benefits of hES cell research are realized given the restrictions of the federal policy, their other main point of emphasis has often been the economic benefits and prestige that are likely to result from attracting hES cell researchers and biotechnology companies to the states they represent [6,8,9]. What these officials have not stressed much, if at all, however, is the potential impact of these initiatives on the problem of unequal biological access as it is likely to arise in their respective states. There seems to be little awareness of this problem among these officials, even though, as we shall see, the initiatives they support could potentially play a significant role in ameliorating its effects.

The final example is the announcements last year by two research groups of their success at "deprogramming" human epithelial cells back to a pluripotent state simply by introducing and expressing four genes [10,11]. These "induced pluripotent stem cells" (iPS cells) are believed to have the same properties as hES cells. If this is true, this method offers the best chance so far of addressing the problem of unequal biological access, and yet, little mention of this possibility has been made in most media reports heralding these researchers' results. Instead, the excitement generated has largely been due to the claims of various scientists, politicians and reporters that these researchers have discovered a way to achieve all of the benefits of hES cell research without the accompanying ethical objections arising from the destruction of embryos.

This paper is motivated by the belief that those of us living in the United States need to begin taking the problem of unequal biological access more seriously than we currently are. hES cell research is rapidly moving towards the point when the first therapies using hES cells will become available to patients. Since most will agree that we have an obligation to ensure that access to these therapies is equally distributed across the general population of the United States, we need to begin taking the steps now to make certain that this obligation is met. Thus, the aim of this paper is to draw attention to the problem of unequal biological access once again with the intent of increasing the pressure on all those involved to openly acknowledge it and to do more than is currently being done to address it.

Specifically, I do the following in this paper. I begin by explaining why there is concern that the problem of unequal biological access will arise in the United States unless deliberate steps are taken to prevent it. Crucial here is the fact that currently researchers almost exclusively derive hES cells from discarded embryos. Since, as I show, it is mostly white Americans who donate these embryos, this makes it highly likely that whatever therapies are developed using these cell lines will benefit white Americans to the relative exclusion of most others in the general population. I next argue that we have an obligation to address the problem of unequal biological access so as to prevent this disparity in access to hES cell-based therapies from occurring. Finally, I lay out four steps that we need to take to solve the problem of unequal biological access. As a part of this discussion, I consider what, besides simply being unaware of it, is the likely reason that the problem of unequal access has not been taken seriously so far. Many seem to think that new methods for generating human pluripotent cells, such as SCNT or the iPS cell technique, will become available that will either circumvent the problem or enable researchers to mitigate its negative effects. I show why there is good reason not to rely on these methods to solve the problem of unequal biological access to the therapies that may arise from hES cell research.

### The problem of unequal biological access: regenerative therapies

The ability of hES cells to become any kind of cell in the human body – what scientists call their "pluripotency" – leads many researchers to believe that they have the potential for two kinds of therapeutic applications. One involves using these cells as the basis of "regenerative therapies," where hES cells are differentiated into specific kinds of tissues for transplant into patients. The goal of those pursuing this kind of application is to develop tissues to treat such diseases and afflictions as Parkinson's, Alzheimer's, diabetes, spinal cord injury, stroke, burns, and heart disease. They also hope eventually to succeed at growing whole organs to alleviate the chronic shortage faced by those needing a whole organ transplant. The other therapeutic application currently being pursued by researchers involves differentiating hES cells into specific tissues that can then serve as models for studying the development, progression and possible treatments of diseases that target these tissues. Besides allowing researchers to study diseases and treatments at all stages of a disease's development, these hES cell-based models would overcome the most significant limitation that arises from using animal models for medical research. Since these models consist of human cells, there is no question of whether findings can be extended to human beings.

The problem of unequal biological access arises in the context of both of these therapeutic applications. Since Faden and her colleagues have already shown in their article how this problem arises in the case of hES cell-based regenerative therapies, my discussion of it in the context of these therapies in this section will mostly summarize their work. Faden and her colleagues, however, have little to say about the problem of unequal biological access as it arises in the context of tissue models. So, my discussion of the problem in this context, which I undertake in the next section, will go beyond what they provide.

Assuming that researchers will be able to coax hES cells to differentiate into the specific kinds of tissues needed for transplant, the main obstacle to the success of hES cell-based therapies is immune rejection. Unless there is a certain type of genetic compatibility between the transplant and the patient, the transplant will be rejected and destroyed by the patient's immune system. More specifically, immune rejection is controlled by genes that code for a kind of protein called the "human leukocyte antigens" (HLA). These HLA proteins are on the surfaces of just about all of a person's cells, and except in abnormal cases, they keep a person's immune system from attacking its own tissues. Each person's immune system recognizes its own HLA protein type and refrains from attacking any tissues that have it. Any tissues identified as having a foreign HLA protein type, on the other hand, are attacked and destroyed. Embryonic stem cells and all subsequent cells derived from them have HLA proteins. Thus, any transplanted tissues created from these cells will be attacked and destroyed by the recipient's immune system if they do not have the same HLA protein type as the recipient. There are only two ways to keep this type of rejection from happening. One is to match transplanted tissues to recipients based on HLA protein type. In cases where such a match is possible, rejection may be avoided altogether or the threat of it may be significantly reduced. The other is to use immunosuppressive drugs to keep the immune system from attacking the transplant. Using the drugs that are currently available, however, is financially burdensome to patients and often causes additional serious health complications. For these reasons, in most cases, obtaining an HLA match is seen as the more desirable of these two options.

The problem with matching HLA protein types, however, is that it proves to be rather difficult to do. First, three sets of genes play important roles in regulating immune rejection- HLA-A, HLA-B and HLA-DR. Second, these genes are highly polymorphic, which means that there is significant variability in these genes among the general population. A variant of a gene is referred to as an "allele." As humans are diploid, having two alleles of every gene, obtaining a complete match in HLA protein types between a donor and a recipient requires matching the two alleles of each of these three HLA genes.

To illustrate just how difficult it is to obtain an HLA match between recipient and donor, Faden and her colleagues draw attention to the registry maintained by the U. S. Bone Marrow Donor program. This registry is a list of over four million donors who have had their HLA-A and HLA-B alleles typed. Faden and her colleagues note that "Due to the high degree of polymorphism in the relevant alleles, even with this enormous pool of donors only 50 to 60 percent of patients who need transplants can find a match" [[1], p. 17].

Further complicating attempts to obtain an HLA match is the fact that HLA expression "tracks with geographical ancestry" [[1], p. 15]. HLA alleles occur with different frequencies within each ancestral group. Alleles common to one group may be much less common in another. As evidence of this, Faden and her colleagues give the example that "the ten most common HLA-A alleles in white Americans are not the ten most common in African Americans, and vice versa" [[1], p. 17]. This fact about HLA distributions complicates matters because it means that success in finding an HLA match will depend on which ancestral groups are represented in the donor pool. Members of groups that are underrepresented in this pool may have significantly greater difficulty finding a match, while individuals from groups that are better represented will find it much easier.

It is this last point that gives rise to the concern that the problem of unequal biological access will arise in the case of hES cell-based regenerative therapies. There is good reason to think based on what we know about the pool of embryos from which existing hES cell lines were derived (i.e., those currently available for use in federally funded research and those that have more recently been created for state or privately funded research) that the genotypes of ethnic minorities are underrepresented and those of white Americans are overrepresented in these lines. The vast majority of these lines were derived from discarded embryos left over from fertility treatments utilizing *in vitr*o fertilization. Studies show that there is significant ethnic disparity in the use of reproductive technologies within the general population of the United States [12-14]. African Americans and Hispanics, for instance, underutilize these services, while white Americans utilize them to a much greater degree. This disparity strongly suggests that any existing hES cell lines derived from discarded embryos came from a pool of embryos that was likely genetically biased in favour of white Americans and that this will continue to be true in the case of any future hES cell lines also derived from this pool. Given what has been said above about the importance of HLA matching, this all boils down to an increased likelihood that white Americans will have significantly greater access to hES cell-based regenerative therapies than everyone else as long as researchers continue to use discarded embryos as the source of their hES cell lines.

Two additional points need to be made here. First, it is true that in the case of organ transplantation the importance of HLA matching has been shown to vary depending on what type of organ is transplanted [15]. For instance, kidneys are sensitive to rejection and a close HLA match is essential to a good clinical outcome. Livers, on the other hand, are less sensitive and obtaining a close HLA match is not regarded as a top priority. Hearts and lungs fall in between. Thus, the extent to which there will be ethnic disparities in access to hES cell-based regenerative therapies if the problem of unequal biological access is not addressed will certainly depend on the type of regenerative therapy that is in question.

Second, immunosuppressive drug therapies can also be used to increase access to hES cell-based therapies in cases where a good HLA match is not possible. However, given that the use of these drugs imposes significant financial burdens and health risks on patients, increasing ethnic minorities' access to regenerative therapies by means of these drugs will not be sufficient for addressing the problem of unequal biological access. Since ethnic minorities would be forced to endure these burdens and risks disproportionately to white Americans, the problem still remains. To address the problem of unequal biological access adequately using immunosuppression, what is needed is a therapy that makes the risks and expense of regenerative therapies in these cases comparable to those that involve good HLA matching. Some recent reports suggest that one therapy, "mixed chimerism," has promise along these lines [16,17]. It involves temporarily suppressing the immune system of the recipient and transplanting bone marrow from the organ donor into the recipient prior to the actual organ transplant. When successful, this therapy creates a chimeric immune system in the recipient, consisting of the recipient's and the donor's immune cells, that does not reject the transplanted tissue. This therapy still has a way to go before its long-term effectiveness and safety is established. However, if these are ever established, this therapy would certainly prove significant for addressing the problem of unequal biological access. Until then, the lack of genetic diversity in the hES cell lines available to researchers must remain a concern.

### The problem of unequal biological access: tissue models

While the use of hES cells for regenerative therapies often receives the most attention in discussions of the potential benefits of hES cell research, many researchers see the more immediate benefits, and perhaps even the more significant ones, as coming from the use of these cells to create "tissue models" for studying the development and possible treatment of various diseases. For instance, one strategy currently being pursued is to use preimplantation genetic diagnosis to genetically screen embryos for particular genetic diseases, derive hES cells from those predisposed for these diseases and then generate diseased tissues using those cells. This then would allow researchers to study the development of the disease from its earliest stages and to test treatments at any point. It is striking what this could someday mean for a disease such as cystic fibrosis, an inherited disease of the mucus glands which causes progressive damage to the respiratory system and chronic digestive problems among others. While researchers know that cystic fibrosis is caused by a mutation in the CFTR gene, they suspect that other factors, both environmental and genetic, influence the disease's progression and are the reason why some with the disease are more severely afflicted than others. Having a cystic fibrosis disease model would give researchers a powerful tool for isolating and identifying these factors.

Another strategy is to use hES cells to develop tissue models that are not diseased for use in testing the effectiveness and toxicity of drugs. For example, this strategy is currently being pursued at the University of Georgia where researchers are looking for ways to combat spinal muscular atrophy or SMA, the number one genetic killer of children under two years old. SMA is caused by a defect in one of the two survival motor neuron (SMN) genes that people have, a set of genes responsible for producing a protein necessary for the proper development and functioning of motor neurons. In children with SMA, the second SMN gene does produce this protein, but not in amounts sufficient to keep the child's motor neurons from degenerating. Researchers at the University of Georgia hope to produce systems of motor neurons from hES cells to test the effectiveness and toxicity of drugs that offer the promise of increasing SMN protein levels [18].

Unlike researchers working towards hES cell-based regenerative therapies, researchers looking to develop hES cell-based tissue models do not encounter the obstacle of immune rejection, since the tissues they generate will not be used for transplants. However, having genetically diverse hES cell lines to work with is still essential for those developing these models.

It is well documented that genetic variability has a role in determining whether a person develops a specific disease. The gene or genes responsible for the disease may be polymorphic and the development of the disease and its severity may depend on which alleles of the gene the person carries. And, often, diseases are "polygenic," meaning that the presence of specific alleles of other genes is necessary for a disease to develop. Many high profile diseases, such as heart disease, cancer and diabetes, seem to fall into this category. It is also well known that the effectiveness and toxicity of drugs often depend on which alleles of specific genes a person has, most often because these variations affect how drugs are metabolized. For instance, a group of enzymes found in the liver and gut called the "cytochrome P450 system" plays an important role in metabolizing many drugs. Each of these enzymes is the expression of an individual gene, and so far, over fifty P450 genes have been identified. Moreover, there are many alleles of each these fifty genes, and a drug's effectiveness and toxicity can vary greatly depending on which allele an individual has [19]. Thus, without genetically diverse hES cell lines there is the real risk that the resulting lack of genetic diversity in the tissue models created from these lines will significantly undermine the ability of researchers to understand adequately the development of diseases and to assess properly the effectiveness and risks of potential treatments of them, as well as accurately assess the potential effectiveness and toxicity of drugs as they are metabolized in the body.

The problem of unequal biological access is specifically a concern in this case because the genetic variations that influence disease development and drug effectiveness can occur with different frequencies among ancestral groups. Cystic fibrosis is an example of a disease of which this is true. It is much more common among white Americans where approximately 1 in 3,200 white newborns are diagnosed with the disease, as compared to African Americans and Asian Americans where roughly 1 in 15,000 and 1 in 30,000 respectively are diagnosed as having it [20]. Another example is prostate cancer where the incident rate in African Americans (248.5 per 100,000 men) is much greater than in white Americans (156.7 per 100,000 men) [21]. While this difference can be partly attributable to such factors as access to healthcare, diet and other lifestyle characteristics, there is growing agreement that differences in allele frequencies between ancestral groups is an underlying cause [22]. The genes underlying the cytochrome P450 system, on the other hand, serve as a good example of genes influencing drug effectiveness whose variants often occur with different frequencies among ancestral populations. For instance, one of these genes, *CYP2D6*, has a role in converting codeine into its active form, morphine. Those who have two nonfunctional alleles of *CYP2D6 *are unable to make this conversion, and thus do not experience the drug's analgesic effect. The frequencies of these nonfunctional alleles vary significantly, ranging from 6 percent in Asian populations to 7 percent in African populations and 26 percent in European populations [20]. Although these are only three of the many examples that could be mentioned, they do illustrate what is at risk if one ancestral group is overrepresented in the hES cell lines used to develop tissue models.

As these examples show, genetic variants responsible for diseases occur in all ancestral groups. Thus, they can show up in hES cells lines derived from discarded embryos (such as those found to be genetically defective following PGD testing) even if one ancestral group is overrepresented in them. However, the significant differences in the frequencies of these variants between ancestral groups will often make their appearance highly unlikely. Hence, if one group is overrepresented in the hES cell lines available to researchers, there is the risk of developing a biased understanding of disease susceptibility and drug efficacy that favors the overrepresented group and increases the risks for those in underrepresented groups. If this overrepresented group does turn out to be white Americans, as there is reason to suspect it will, then white Americans will receive a disproportionate share of the benefits of hES cell-based tissue models, while minority ethnic groups will be forced to endure a disproportionate share of the risks.

### Why we have an obligation to address the problem of unequal biological access

Recognizing that hES cell research as it is currently being pursued – that is with discarded embryos as the primary source of hES cell lines – will likely lead to disparities in access benefiting white Americans to the relative exclusion of other ethnic groups in the general population will be enough to convince some that we have an obligation to address the problem of unequal biological access. Others, however, will require more to be convinced. Thus, it is worth giving a general sense of how this problem fits into the broader context of health and healthcare disparities in the United States and how it is a further extension of those disparities.

That there are disparities in healthcare and health in the United States is well documented. For instance, health insurance is the gateway to the healthcare system, and yet the U.S. Census Bureau reported in 2005 that as many as 46.6 million people in the United States lack it [23]. Moreover, ethnic minorities are much more likely to be without health insurance than white Americans. In 2005, 11.3 percent of non-Hispanic whites lacked health insurance, while 19.6 percent of African Americans and 32.7 percent of Hispanics did not have it [24]. The impact of not having health insurance on people's health and financial viability cannot be overemphasized. Those without it are less likely to receive preventative care and thus to be in advanced stages of a disease once they are examined by a physician. They also tend to be sicker upon being admitted to a hospital and thus more likely to die after being admitted. In addition, even though those without health insurance tend to be poorer, they often have greater out of pocket expenses and have higher rates of bankruptcy due primarily to their medical expenses [25].

But studies also show that even when ethnic minorities have health insurance they often receive less access to healthcare than do white Americans. They are, for instance, less likely to be screened for cancers, diabetes and cardiac risk factors. They are also less likely to receive prenatal and maternal care resulting in greater postnatal complications for newborns and mothers and higher infant mortality rates. And, much too often, the treatments for many diseases have significantly lower success rates for ethnic minorities than for white Americans [25].

This last point draws attention to the disparities experienced by ethnic minorities in medical research. The history of this research is littered with examples where the interests of these groups have been intentionally violated in favor of the "interests of society," which most often meant the interests of white Americans. The classic case, of course, is the Tuskegee Syphilis Experiment where beginning in 1932 and continuing until 1972 African American men were intentionally denied effective treatments for syphilis so that the effects of the disease on them could be compared to its effects on white Americans (even though no similar experiment was ever started on white subjects). Other more recent cases may not involve such blatant, intentional mistreatment, but the fact remains that they are examples where the interests of minority ethnic groups have not been addressed. For example, one explanation for why some treatments are less effective for ethnic minorities is that they are less likely to be included as subjects in clinical trials. This may be partly due, as is often claimed, to their unwillingness to volunteer for clinical trials because of their distrust of the medical community; however, recent studies suggest that this lack of participation is more often due to researchers failing to reach out to members of these groups because of pre-existing beliefs about the difficulties in recruiting them, the higher costs in doing so and the increased likelihood of their dropping out of trials [26].

Although much more can be said here, this gives a sense of the context in which the problem of unequal biological access needs to be understood. Currently, there is every expectation that hES cell research will radically change how some of humanity's most devastating diseases and afflictions are treated. The success of hES cell-based therapies will mean significant improvements in the overall health and life expectancies of those who have these diseases and afflictions. However, what the problem of unequal biological access brings to our attention is that some minority ethnic groups, those very same groups who have traditionally been subjected to health and healthcare disparities in this country, are likely to receive significantly less access to these therapies because the hES cell lines available to researchers lack the genetic diversity to ensure that this access is more equally distributed across the general population.

But there is more. This lack of genetic diversity in these hES cell lines is itself a direct result of ethnic disparities in access to healthcare. I have already mentioned the extent to which ethnic minorities underutilize the reproductive technologies that currently supply researchers with embryos. The reasons most often given for this underutilization all point to the effects of past and current discrimination experienced by these groups. Ethnic minorities often lack the financial resources to pay for these reproductive technologies, and when they do have these resources it is common for them to face barriers impeding their access to these technologies. Too often, ethnic minorities are not informed of the availability of these technologies, do not receive the necessary referrals from their primary physicians, and are even denied access because of ethnic discrimination [12].

Most of us agree that as members of a society that has a history of ethnic discrimination, we have an obligation to do whatever we can to alleviate the negative outcomes of existing disparities and to prevent future ones from occurring. One of these negative outcomes is the problem of unequal biological access. Thus, we have an obligation to take whatever steps we can to address this problem so as to prevent any future disparities that are likely to result from it. My aim in the rest of this paper is to sketch in general terms what these steps are.

### Step one: we must openly acknowledge the problem of unequal biological access

At the beginning of this article I mentioned four examples to illustrate how little public attention is being paid to the problem of unequal biological access. This lack of attention may really be because people are unaware of the problem in spite of the attempts by Faden and her colleagues (and others) to draw attention to it. If so, it is obviously my hope that this paper will increase awareness of the problem and motivate more people to do more about it. However, I think there are two other reasons why the problem of unequal biological access has received insufficient attention up to this point. I discuss these in the next two sections.

The debate over hES cell research is highly politicized. As a result, each side can have the tendency to emphasize whatever it deems to be to its advantage, while ignoring whatever is deemed otherwise. I suggest that one possible reason why the problem of unequal biological access has not received the attention it deserves is that often neither side in this debate sees much to gain by drawing attention to the problem, and perhaps even much to lose if it does so.

This can be shown by focusing on what is at stake for each side in the debate over the current federal policy regulating funding for hES cell research. To qualify for funding an hES cell line must have been created from a donated, discarded embryo prior to President Bush's announcement of this policy on August 9, 2001. Proponents of this policy regard it as a compromise between those who want hES cell research to proceed and those who demand that it be stopped because of its destruction of embryos. Acknowledging the problem of unequal biological access would, however, put these proponents of the policy in a difficult place. Although President Bush stated in his announcement that "more than 60 genetically diverse stem cell lines already exist" [27] that would qualify for funding and would eventually become available to researchers, acknowledging the problem of unequal biological access would require admitting that the lines available to researchers are almost certainly not as genetically diverse as the President suggested. This is a possibility made even more likely by the fact that currently only twenty-one qualifying lines are actually available. But, of course, acknowledging the problem of unequal biological access involves more than just recognizing this lack of genetic diversity. It also means admitting that because of this lack of diversity the federal policy, albeit unintentionally, increases the likelihood that disparities in access to the benefits of hES cell-based therapies favoring white Americans will occur. Again, this is not a place where proponents want to find themselves. They do not want to appear to be supporting a policy that at best blocks attempts to avoid these disparities, and at worst, exacerbates them.

It is more difficult to see why those who oppose the federal policy because it is too restrictive would be reluctant to acknowledge the problem of unequal biological access. One would think that doing so would pressure proponents of the policy to support attempts to loosen its restrictions. The situation becomes clearer, however, once it is recognized that replacing the federal policy with the one that most of these opponents endorse is unlikely to make much progress towards addressing the problem of unequal biological access. What these opponents often seek is a policy along the lines of the Stem Cell Research Enhancement Act that allows federal funding to support research on new hES cell lines, but only if they are derived from donated, discarded embryos. However, even though such a policy change would mean the creation of new hES cell lines, and perhaps even a significant number of them, what can be concluded about the likely genetic diversity of these discarded embryos based on what we know of who has access to reproductive technologies gives little reason to expect that these extra lines will make access to the benefits of hES cell research much more equal. Thus, for these opponents of the federal policy, focusing attention on the problem of unequal biological access comes with the risk of exposing a serious shortcoming of their own policy.

I do not see how refusing to acknowledge the problem of unequal biological access for the reasons above can be defended. Our obligation to address existing healthcare disparities and to prevent future ones from occurring amounts to an obligation to formulate public policies that ensure that all in the general population have an equal opportunity to access the benefits of publicly funded medical research. It also certainly means that we have an obligation to correct any existing policies that are likely to lead to disparities in this access. We are not released from these obligations just because the public policies in question pertain to hES cell research.

The second possible reason for why the problem of unequal biological access has not received much attention is less political and perhaps even more understandable. Many who are aware of the problem may believe that there is no need to draw attention to it because by the time hES cell-based regenerative therapies and tissue models are clinically viable, researchers will no longer be relying on hES cell lines derived from discarded embryos to develop them. Instead, the development of new techniques such as SCNT, or the one used to obtain human iPS cells, will enable researchers to produce pluripotent stem cells that are genetically specific to individual patients. This would eliminate the problem of unequal biological access, since regenerative therapies and even tissue models could be tailored to each patient who needs them.

There are two points to be made here. First, researchers are already moving forward with developing tissue models using hES cell lines that have been derived from discarded embryos. The work being done at the University Georgia to develop motor neuron models to test drug treatments for SMA is just one example. There are many other research laboratories across the country pursuing similar projects. At this time, there is every reason to expect that some of the treatments being tested using these models will become available to patients in the near future. Also, two companies – Geron Corporation and Advanced Cell Technology – have recently sought Food and Drug Administration approval to begin Phase I trials testing regenerative therapies that were developed using hES cells from discarded embryos [28]. The point is that hES cell research is already on the brink of moving into a therapeutic stage. As I see it, we can ill afford not to acknowledge the problem of unequal biological access or to wait to address it until either SCNT or the iPS cell technique (or some other one) is available, if either one ever is. For one thing has become clear, once ethnic disparities in access to healthcare exist, they are extremely difficult to address.

Second, even if SCNT or the iPS cell technique is successfully developed, neither is likely to fulfill all that it promises. Both will be able to provide researchers with patient-specific stem cells, but it is unlikely that researchers will be able to use either of these techniques to produce stem cells for patients on demand. Thus, neither of these methods will likely enable researchers to avoid the problem of unequal biological access by ensuring that all in the general population who might benefit from hES cell-based therapies will have access to them. Neither method is likely to make "full access" to the benefits of hES cell research a reality, at least not in the near future. Recognizing this and openly acknowledging it is the second step we need to take to address the problem of unequal access.

### Step two: we must acknowledge that full access for everyone is not possible at this time

Being able to provide all patients with hES cell-based therapies specific to their own genotypes would, of course, be the best possible solution to the problem of unequal biological access. At least in principle no one in the general population would be denied access to these therapies for biological reasons alone. There would be no need to worry about HLA matches in the case of regenerative therapies and tissue models could be developed for each patient so that drug treatments could be tested for efficacy and safety. This degree of access is most certainly not achievable as long as researchers rely on discarded embryos as the source of hES cell lines, but it does seem achievable, at least at first glance, if either SCNT or the iPS cell technique (or both) is successfully developed. But is it reasonable to expect either of these techniques to make possible this degree of access?

SCNT is a technique where a nucleus from an individual's cell is inserted into an oocyte that has had its nucleus removed, and the re-nucleated oocyte is then stimulated into developing. Once the resulting embryo develops into a blastocyst, hES cells can then be harvested from it using the method for deriving these cells from discarded embryos. Since the nucleus used can be taken from any patient's somatic cell, this technique offers a way to derive hES cells that are genetically specific to patients.

In the United States, researchers at a number of institutions are currently working towards developing SCNT into a reliable method for obtaining hES cell lines. Harvard University, The University of San Francisco, Stanford University, and Memorial Sloan-Kettering Cancer Center, among others, all have active research programs in this area supported by state and private funding. Researchers in other countries, such as Japan, South Korea, Great Britain and Australia, are also working towards developing this technique. While these researchers have yet to succeed at obtaining hES cells using SCNT, most continue to believe that it is only a matter of time before it will be done.

But even if SCNT is developed into a reliable method for generating patient-specific hES cells, there are good reasons to think that researchers will not be able to use it to provide hES cells on demand for every patient. First, there is the lag time that will exist between when patients are diagnosed as requiring an hES cell-based therapy and when the hES cell lines genetically specific to them will become available. It is doubtful that most patients will be able to wait this long for their treatments to begin. Second, there is the almost certain high cost of using SCNT to develop patient-specific hES cells lines. We can hardly expect sufficient financial resources to be allocated to cover the expense of using SCNT for all patients needing hES cell-based therapies. We might also rightly question whether this is the best use of the limited financial resources available for ensuring equitable access to healthcare. Further complicating these matters is that research involving hES cells obtained using SCNT would not qualify for funding under the current federal policy because of the harm it does to embryos. Finally, third, there are numerous ethical and practical barriers to using SCNT on such a wide scale. For instance, SCNT would create embryos with the specific purpose of harvesting hES cells from them. Many people believe that there is a clear moral difference between using discarded embryos for hES cell research and intentionally creating embryos specifically for this purpose. In fact, many would withdraw their support of this research in this latter case. There are also the important questions that arise due to the reliance of this method on donated ova. Will women be willing to donate the number of ova that the use of this technique on such a wide scale would require? If not, should they be paid for donating them? How likely is it that women will be coerced into donating their ova? Will the general public tolerate and support the use of alternative sources of ova, for instance, those obtained from cows or rabbits [29,30]? For all these reasons, we should be hesitant to rely on SCNT as the method for achieving full access.

The iPS cell technique, on the other hand, is a more promising option. Because iPS cells are derived directly from human epithelial cells, they are a source of patient-specific pluripotent stem cells that does not require destroying a single embryo or using a single ovum. In this way, they bypass all of the ethical objections that can be raised against SCNT. Research both to produce these iPS cells and to develop therapies from them would also qualify for federal funding, and given the claims by researchers that the iPS cell technique is relatively easy to use, much easier than SCNT, it is likely that significantly less financial resources will be needed to produce patient-specific cells on demand.

However, in spite of these advantages, it would be a mistake to assume at this point that the use of the iPS cell technique will make full access possible, at least in the near future. This technique for obtaining these stem cells is in the earliest stages of development and is still a long way from being used to derive iPS cells for therapeutic applications. There is still the chance that human iPS cells will turn out not to be pluripotent or that the technique for producing them cannot be perfected to the point where it can be safely and reliably used. While the very recent report [31] by one research group of their success at using the iPS cell technique to turn skin cells from an 82 year-old ALS patient into pluripotent stem cells that then differentiated into motor neurons with her genetic makeup does reinforce its viability for generating patient-specific pluripotent stem cell lines that could then be used for disease models, most researchers continue to believe that tissues generated from iPS cells are unsuitable for transplantation because one of the genes employed to induce dedifferentiation and the retroviruses used to insert the genes into somatic cells have oncogenic properties [31,32]. Researchers continue to stress that the use of iPS cells for regenerative therapies will need to be delayed until alternative methods for dedifferentiating cells become available [31,32]. But even if this technical obstacle is overcome, there is also the issue that until the technique is perfected to the point where it is actually easy to use, and its initial costs are significantly reduced, it remains unlikely that the iPS cell technique will be made available to all patients who might benefit from it. In fact, one has to be concerned that there will be disparities in access to iPS cell based therapeutic applications as a result of access being limited, at least initially, to just those patients who are able to pay for it themselves. In addition, there is the worry about lag time between diagnosis and availability of treatment. As with SCNT, it will likely require some months to obtain iPS cells and to develop the appropriate tissues from them.

However, it would also be a mistake to conclude from this that the successful development of iPS cells would not be an important advance towards solving the problem of unequal biological access. Once it is recognized that this problem is unlikely to be solved in the near future by pursuing a strategy of providing full access, the goal has to become one of ensuring that whatever access is possible to achieve is more equally distributed across the general population. This means acknowledging that Faden and her colleagues were right in arguing that what is required is a strategy that deliberately aims at developing a bank of stem cells lines available to all researchers that more accurately mirrors the genetic diversity of the general population. Such a bank would not only work to address the problem of unequal biological access, but would also help with the lag time issue and ensure that all researchers and clinicians have an adequate supply of pluripotent stem cell lines available to them. For reasons already discussed, such a bank is unlikely to be established if researchers continue to rely on donated embryos to provide hES lines with the required genetic diversity. Instead, they will need a method that allows them to create pluripotent stem cell lines with targeted genotypes. If iPS cells can be reliably produced, they will offer the most practical and least controversial way yet for generating these lines and for populating a genetically diverse and public stem cell bank. It is here that the real value of iPS cells potentially lies, and it will be significant even if the use of these cells is limited to disease models.

### Step three: we must publicly decide what counts as equal access

But even if we agree that addressing the problem of unequal biological access requires a public policy of deliberately establishing a public and genetically diverse bank of hES cell lines, or pluripotent cell lines generated using alternative methods, the question remains as to which genotypes to target for inclusion in this bank. We do have choices here. We can, for instance, aim to maximize coverage among all those in the general population. We can also place an emphasis on treating all in the general population "equally" by adopting a lottery system where all have a chance to have their genotypes included. Still another option is to work towards ensuring that all ethnic groups in the general population are in some way equally represented in the bank so that no group has a disproportionate advantage. What I have said in this essay up to this point makes it obvious that a method along the lines of this third option would be the one I support, and in this I am more or less in agreement with Faden and most of her colleagues. In their paper, these authors considered all three of these options and rejected the first two primarily because pursuing them would increase the risk that ethnic minorities would experience disparities in access to the benefits of hES cell-based therapies. They chose the third option of distributing access equally among all ethnic groups because it mitigates this risk, while even doing some work towards correcting already existing disparities in health and healthcare. Of course, this option faces its own set of questions that will need to be answered, not the least of which will be how to determine which genotypes within ethnic groups should be included in the bank, which is not to say that Faden and her colleagues have nothing important to say in response to such questions.

However, my aim here is not to defend a particular method for selecting the hES cell lines to be included in an public stem cell bank. Such a defense would be beyond what I can accomplish here. Instead, I want to emphasize how important it is that the right process be used to choose this method.

We need to keep in mind that regardless of which method is ultimately used to select the hES cell lines for inclusion in a stem cell bank, some number of people who might potentially benefit from hES cell-based therapies will be denied access to them simply because their genotypes are not represented in the lines selected. This fact makes it important that our choice of a method be the product of public deliberation of the various alternatives. Such deliberation serves a number of critical purposes in this case. For one, it allows for the critical assessment and correction of proposals, which not only increases the probability of success, but also makes the justice of the strategy chosen more likely, since hidden biases will be uncovered and undesirable consequences identified. It also allows all those who are affected by the decision to offer input into the decision making process, which is something justice in this case also seems to require. Given what is at stake, all those affected should have an opportunity to shape the decision so that their interests are addressed, and if the decision goes against their interests, to understand the process that led to that decision. This last point must not underappreciated. A public and genetically diverse hES cell bank will be much easier to establish if there is wide public support for it including from those whose genotypes will not be represented in it. As such, people need to be convinced of the need for such a bank, its potential for success, and its fairness.

The case for the importance of such a process becomes even stronger once we recognize that much of what is wrong with the National Stem Cell Bank could have been avoided if a process like this one had been followed. The purpose of this bank is to facilitate research on the twenty-one hES cell lines that qualify for federal funding. Certainly one concern that many researchers have raised about the National Stem Cell Bank is that this number of lines are just too few to meet their needs. Many have also pointed out that some of these lines have changed genetically or do not differentiate equally well into all tissue kinds, and thus are limited in their suitability both for regenerative therapies and tissue models [33]. Additionally, it is well known that all of these lines were derived using a method requiring mouse feeder cells, and thus any regenerative therapies utilizing tissues created from them carry the risk of transferring mouse viruses to humans [34]. And, of course, we must question the extent to which minority ethnic groups are represented in these lines. After all, each of these lines was derived from a discarded embryo, and we have already seen why this should lead us to suspect that white Americans are overrepresented in these lines.

The point is that these problems with the National Stem Cell Bank are largely due to the process that led to its creation. The President announced a policy that was not subjected to rigorous public debate prior to its being announced. As a result, serious shortcomings in the policy were not identified and corrected, which in turn has led to a national stem cell bank that is at best ineffective and at worse unjust. And the failure of the President to give those who are most likely affected by this policy a chance to shape it through their input has led many to resent it deeply. Whether rightly or wrongly, they see it as something forced upon them by a President who is either acting on his own religious beliefs or out of a desire to appease his own political constituencies.

So if I am right about the process we need to pursue for selecting a method for choosing hES cell lines for inclusion in a public and genetically diverse stem cell bank, the third step we need to take to address the problem of unequal biological access is clear. We need to begin the process of publicly deliberating about the various methods that seem to be plausible. This will require the contribution of those with expertise in ethics and law to clarify what justice and fairness mean in this case. It will also require the efforts of those with expertise in the biology of stem cells, in tissue transplantation, in disease genetics, among others, to provide the information necessary for informed positions and choices. It also will require politicians to recognize the importance of this debate, to allocate the funding necessary for its taking place, and to refrain from co-opting it for their own political agendas. Finally, it will necessitate efforts on everyone's part to ensure that the general public has opportunities to participate in this debate and to be kept informed of its progress and conclusions.

I do not want to suggest that any of this will be easy to accomplish. Obviously, it is extremely difficult to initiate and to sustain the kind of deliberative process I am describing here. It is also unlikely that we will ever reach a consensus on the method to use for selecting the hES cells to populate the bank. Almost certainly, a public entity, such as the NIH, will need to make the decision as to which method to pursue. But, I do think it is right that how this decision is reached will be crucial to garnering wide support for creating a public stem cell bank that is perceived by most in the general population as being just, and thus, that it is worth our expending the effort to do all we can to make sure that this decision is reached in the right way. We do have mechanisms in place already to reach decisions like this one. The President's Council on Bioethics, for instance, is already charged with exploring the ethical and policy questions related to bioethical developments, which includes providing a forum for national discussions of these developments. While it is true that such councils can be unduly influenced and manipulated by political pressure, as some have claimed the current President's Council has been, a council that successfully resists this pressure would prove invaluable to ensuring that the process of selecting a method for populating an hES cell bank is just to all.

### Step four: we need to establish a public and genetically diverse stem cell bank

There is reason to be concerned that this last step will not be completed even if the other three are. As we have seen, it would be unreasonable to expect that a genetically diverse stem cell bank can be established using hES cell lines obtained from discarded embryos. The hope, of course, is that a morally uncontroversial technique, such as the one used to obtain iPS cells, will emerge and enable researchers to create lines with targeted genotypes so as to populate the bank. And, as has already been noted, recent, but still isolated reports, suggest that the iPS technique may be viable for creating a bank for tissue models, although their suitability for generating safe regenerative therapies remains in doubt. But, there is still the chance that no alternative technique for producing pluripotent stem cells will prove to be reliable or will be adequately developed in the near future. If this is how things turn out, researchers working towards developing the bank will need to solicit gametes from donors who have targeted genotypes, intentionally create embryos from these gametes, and harvest stem cells from them. This could lead many, and specifically politicians charged with allocating public funds, to withdraw their support for creating the bank because of their moral objections to creating embryos just to destroy them.

There are at least two things to say here. First, it could turn out that public opposition to the use of embryos in this way may not be as severe as might be anticipated. One result of the lack of awareness of the problem of unequal biological access among politicians, bioethicists, the media and the general public is that there has not been much of a discussion of whether intentionally creating embryos and harvesting hES cells from them can be justified as a way of addressing this problem. Thus, it remains an open question whether most would continue to oppose this use of embryos in face of the potential disparities the problem of unequal biological access identifies. It is possible that many would be willing to support (albeit reluctantly) this use of embryos to establish a stem cell bank, if its use were limited to supplementing hES cell lines derived from donated embryos.

Second, if morally uncontroversial methods for populating a stem cell bank fail to develop, and if it turns out that we are still unwilling to create embryos with targeted genotypes so that we can harvest hES cells from them, there are other things that can be done that would at least mitigate the effects of the problem of unequal biological access. For instance, it would help significantly if there were a national registry of all the hES cell lines that are publicly available, both those that qualify for federal funding and those that do not, and one that provided the information necessary for assessing the genetic diversity of these lines. If agreement could be reached on how much of the general population the hES cell lines available to researchers need to cover to achieve just access to hES cell-based therapies, such a registry would allow researchers to identify where coverage is lacking and to use private or state funds to create new hES cell lines to fill in the gaps. Even without this agreement, a registry of this kind would allow researchers to develop new lines from underrepresented ethnic groups. The registry could also be combined with any international registries that include the necessary information for assessing the genetic diversity of the hES cell lines they list, which would potentially increase coverage of the general population.

How such a registry would be funded depends on how the federal policy regulating hES cell research funding is implemented by the NIH. Currently, the NIH requires that research done with hES cell lines that do not qualify for federal funding be kept completely separate from research done with those lines that do qualify. This has led to the creation of duplicate labs at some universities and research institutions [35]. Given this interpretation of the federal policy, it is unclear whether the use of federal funds to create a national registry that includes those lines that do not qualify for federal funding would be permitted by the NIH. The fact that NIH Stem Cell Registry currently lists only those hES cell lines that qualify for federal funding [4] suggests that it might not. If, in the end, the registry cannot be created and maintained using federal funds, it will need to be funded through state or private sources.

In any case, what is clear from the discussion in this paper is that those of us in the United States must be responsive to the concern that the problem of unequal biological access may arise in our country. We cannot allow ethnic disparities in access to hES cell-based therapies to occur. This means that we need to be more aware of the problem as hES cell research progresses towards providing these therapies and to be more committed to ensuring that access to them is fairly distributed among the general population. This leaves unanswered whether we have an obligation to those in other countries to ensure their access to hES cell based therapies. While important, addressing this issue lies beyond the scope of this paper. But it is intriguing to consider whether the next step should be an international stem cell bank [36].

## About the author

Mark Moller PhD is Assistant Professor of Philosophy at Denison University. He is interested in the ethical implications of stem cell policy.

## Competing interests

The author declares that he has no competing interests.

## Authors' contributions

MM is responsible for the entire manuscript.
